# Artificially Reared *Salmo trutta* Fry in a Natural Environment: Growth and Fitness Compared to Wild Specimens

**DOI:** 10.3390/biology15080630

**Published:** 2026-04-16

**Authors:** Vytautas Rakauskas, Simonas Račkauskas, Danguolė Montvydienė, Živilė Jurgelėnė, Eglė Šidagytė-Copilas, Vesta Skrodenytė-Arbačiauskienė, Saulius Stakėnas, Tomas Virbickas

**Affiliations:** 1 Laboratory of Fish Ecology, State Scientific Research Institute Nature Research Centre, Akademijos Street 2, 08412 Vilnius, Lithuania; simonas.rackauskas@gamtc.lt (S.R.); vesta.skrodenyte@gamtc.lt (V.S.-A.); tomas.virbickas@gamtc.lt (T.V.); 2 Laboratory of Ecotoxicology, State Scientific Research Institute Nature Research Centre, Akademijos Street 2, 08412 Vilnius, Lithuania; danguole.montvydiene@gamtc.lt (D.M.); zivile.jurgelene@gamtc.lt (Ž.J.); 3 Laboratory of Evolutionary Ecology of Hydrobionts, State Scientific Research Institute Nature Research Centre, Akademijos Street 2, 08412 Vilnius, Lithuania; egle.sidagyte@gamtc.lt

**Keywords:** sea trout, fish growth, fish stocking, artificial rearing, fish health, gut bacteria

## Abstract

The decline of salmonid stocks in the Baltic Sea region is a matter of serious concern, prompting many countries to implement widespread stocking of artificially reared individuals to restore or enhance populations. While such interventions are intended to be beneficial, their efficacy remains a subject of ongoing debate. Artificially reared fish often face challenges in adapting to natural environments and may struggle to compete with wild counterparts, potentially leading to reduced growth rates and diminished overall fitness. This study evaluated the growth and physiological condition of naturally hatched versus artificially reared *Salmo trutta* juveniles during their first two years of life, prior to smoltification and seaward migration. The results demonstrated that stocked juveniles exhibited significantly slower growth, a higher incidence of fin damage, and a greater abundance of cultivable gut bacteria compared to wild individuals. Conversely, no significant differences were observed in blood parameters. Such growth retardation suggests potential difficulties in adaptation and recruitment. Consequently, while the release of artificially reared *S. trutta* fry facilitates the restoration of extinct populations, its capacity to enhance existing stocks within Baltic Sea riverine ecosystems may be limited.

## Simple Summary

The global decline of fish stocks is a matter of significant concern, leading to the widespread practice of stocking artificially reared fish across many countries. However, such individuals often face challenges in adapting to natural environments and may fail to compete effectively with their wild counterparts, potentially resulting in reduced growth rates and diminished overall fitness. This study demonstrates that artificially reared and stocked juveniles exhibited slower growth and sustained more frequent fin damage compared to wild individuals. Such growth retardation may indicate difficulties in the adaptation and recruitment of stocked fish. Consequently, while the introduction of artificially reared sea trout fry facilitates the restoration of extinct populations, its potential to enhance sea trout stocks within Baltic Sea riverine ecosystems may be limited.

## 1. Introduction

Global fish populations are facing an unprecedented decline driven by a combination of overfishing, illegal fishing practices, pollution, climate change, and habitat degradation [1]. These factors are similarly impacting sea trout, *Salmo trutta* (Linnaeus, 1758) stocks within the Baltic Sea catchment [2]. Although *S. trutta* was assessed as “Least Concern” for the IUCN Red List of Threatened Species in 2022, the report highlights localised subpopulation declines and projects significant future population reductions due to climate change [3]. Despite its current conservation status, S. trutta remains a species of significant ecological and economic importance in the Baltic region. This importance has necessitated widespread stocking programmes using hatchery-reared fish to support and restore wild populations [2,4,5]. In the Nemunas River basin, the fourth largest tributary of the Baltic Sea, a notable decline in *S. trutta* populations has also been documented [6]. Consequently, significant efforts have been undertaken in Lithuania since the late 20th century to supplement wild *S. trutta* stocks through artificial propagation and stocking [7].

While artificial stocking of *S. trutta* offers potential benefits, the practice may entail adverse effects that necessitate careful evaluation. For instance, hatchery-reared fry often experiences significant challenges in adapting to natural environments post-release [8,9,10,11,12,13]. These individuals may be outcompeted by wild conspecifics for essential resources, such as food [14] and optimal habitats [15]. Such competitive disadvantages can lead to suboptimal growth rates and diminished physiological condition compared to wild populations, potentially jeopardising their initial seaward migration. Recent research underscores that interactions between hatchery-reared and wild salmonids are complex and highly context-dependent. A global synthesis [16] demonstrated that hatchery-origin fish frequently exhibit reduced fitness and may negatively impact wild populations through both genetic and ecological mechanisms. Furthermore, recent reviews highlight that the efficacy of stocking varies considerably based on environmental conditions, genetic lineage, and release strategies [17,18]. Accumulating evidence also suggests that early-life rearing environments profoundly influence behavioural and physiological ontogeny, which ultimately dictates post-release performance. To date, there remains a critical knowledge gap regarding the comparative growth rates and overall fitness of hatchery-reared versus wild *S. trutta* fry within natural stream habitats during the high-mortality period preceding their first migration. Addressing this gap is of significant fundamental and applied importance for fisheries management.

In Lithuania, *S. trutta* produced in state-owned hatcheries are primarily released at the fry stage. The underlying premise is that early natural selection in riverine environments ensures that only the individuals best adapted to wild conditions will successfully reach the smolt stage and migrate to the Baltic Sea. However, the survival rate of artificially reared fry to smolt is remarkably low, with data from the International Council for the Exploration of the Sea (ICES) indicating an average survival rate of approximately 3%. It is generally assumed that those individuals who do reach the smolt stage will exhibit post-smolt survival rates and homing abilities comparable to their wild counterparts. Augmenting adult *S. trutta* stocks with fitter individuals theoretically increases the probability of successful natural reproduction and benefits recreational fisheries [19]. Despite this potential, empirical data regarding the physiological condition of hatchery-reared *S. trutta* parr following their growth in natural environments in Lithuania remain scarce [20,21,22].

This study aimed to assess and compare the growth and physiological condition of wild and hatchery-reared *S. trutta* juveniles throughout their first two years of life, preceding smoltification and seaward migration. The investigation was conducted in two adjacent tributaries of the Siesartis River, which represents one of the most critical salmonid habitats in Lithuania.

## 2. Materials and Methods

### 2.1. Study Sites

**Figure 1:**
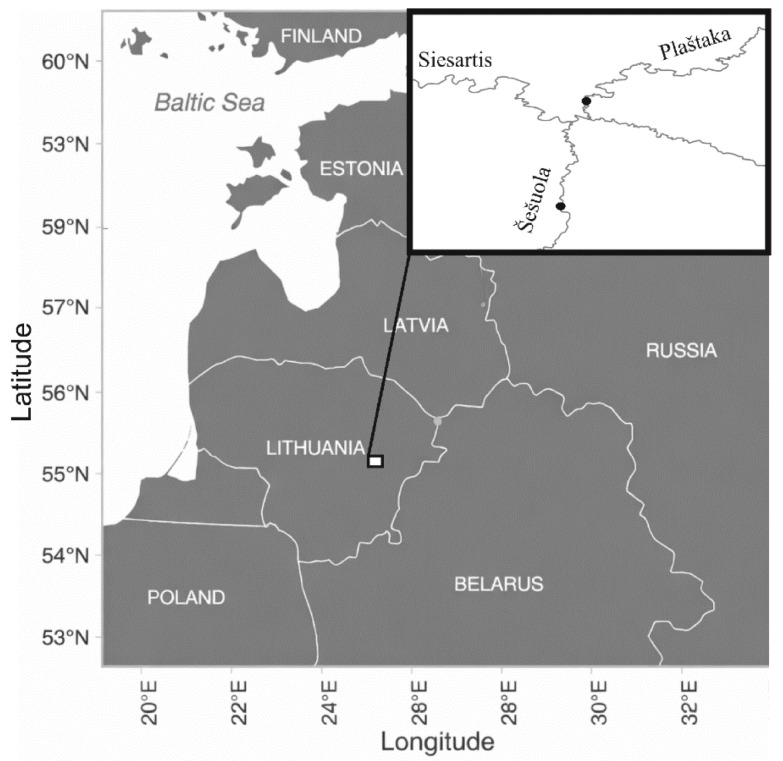
Study sites in the Plaštaka and Šešuola streams. Black dots show artificially reared *S. trutta* stocking places.

The study was conducted in the Šešuola and Plaštaka streams, both of which are tributaries of the Siesartis River within the Nemunas River basin (Figure 1). These watercourses are situated in a hydrological region characterised by predominantly groundwater-fed systems, which account for approximately 40% of the total annual runoff [18]. Geographically and hydrologically, the two streams are highly comparable (Table 1), discharging into the Siesartis River within 0.4 km of each other (Figure 1). In their lower reaches, the streambed substrate is dominated by gravel and pebbles, providing suitable spawning and nursery grounds. The hydromorphology of the Šešuola Stream remains largely natural, although it is channelized between 14 and 10 km from the mouth and obstructed by a dam at the 3 km mark. In contrast, the Plaštaka Stream retains a completely natural hydromorphological state. No known point or diffuse sources of pollution affect the water quality in either catchment. According to national monitoring data, both streams consistently meet the criteria for “good ecological status”, thereby providing optimal habitats for the local wild *S. trutta* population.

### 2.2. Water Temperature

Water temperatures in the investigated streams were monitored using TG-4100 temperature loggers, which were positioned in close proximity to the *S. trutta* stocking sites in 2019, 2020, and 2022. The loggers remained submerged from May (coinciding with the stocking period) through November (the final sampling phase) each year. Measurements were recorded at 30 min intervals daily throughout the three-year study period to capture fine-scale thermal fluctuations.

### 2.3. Fish Stocking

The *S. trutta* fry used for river stocking were obtained from the state salmonid hatchery. In each study year, all artificially reared individuals were derived from a single breeding pair (one male and one female), with a different wild spawner pair collected annually from the Siesartis River to minimise within-year genetic variation. The *S. trutta* fry used for stocking were reared under controlled hatchery conditions in flow-through systems supplied with aerated freshwater. Water temperature was maintained within the range of approximately 12–15 °C during early developmental stages and increased to 16–20 °C during juvenile rearing. Dissolved oxygen concentration was maintained above 6 mg/L, and pH ranged between 6.5 and 7.5. Fish were reared at densities typical for salmonid hatcheries, initially up to approximately 3000 individuals m^−2^ during early juvenile stages and gradually reduced to 1000–1200 individuals m^−2^ as fish grew. Survival rates during rearing typically exceeded 70–80%. Feeding during early ontogeny consisted of live feed (e.g., *Artemia nauplii*), followed by a gradual transition to commercial starter diets, with feeding performed multiple times per day to ensure optimal growth.

To ensure the separation of artificially reared and wild, naturally hatched specimens throughout the experiment, all stocked *S. trutta* individuals were treated with Alizarin Red S (Sigma-Aldrich, St. Louis, USA). ARS dyeing is one of the most widely used methods in practice [24,25,26]. Each study year, up to 4000 *S. trutta* fry were marked with ARS using the technique described by Caudron and Champigneulle [27]. The fry was immersed in a 100 mg/L ARS solution for three hours. During immersion, the fish were kept in a 0.6 m^3^ tank filled with 400 L of aerated deep-well water. After dyeing, all fish were transferred to 1 m^3^ flow-through tanks (minimum flow rate: 1 L per g of wet body mass per day), which were half-filled with aerated deep-well water, for a two-day acclimation period before stocking. All animal procedures were carried out in accordance with Directive 2010/63/EU and the Guidelines for the Care and Use of Laboratory Animals. No mortality of *S. trutta* fry exposed to ARS dye was observed during the marking procedure.

Stocking of the Šešuola and Plaštaka streams occurred in May 2019–2022. Prior to each stocking, a subsample of 25 released *S. trutta* fry was measured and weighed. The detailed numbers, sizes, weights, and release dates of the stocked *S. trutta* fry in each river are presented in Table 2. The fry was released during the second half of May, which minimised the duration of fish captivity and the harmful domestication-related effects [28,29]. All fish stocking activities were performed in the same sections of the streams and followed the same salmonid stocking guidelines used by the State Fisheries Service. In both streams, the fry was evenly distributed along a 100 m stretch located upstream from the stream mouth (see Figure 1 for the detailed stocking locations).

### 2.4. Fish Assemblages

An assessment of fish assemblages in rivers was performed in September 2022. The sampling was performed using battery-powered electric fishing gear (Samus Special Electronics, Samus-725 mp). Fishing was performed up to a 450 m stretch of each river downstream from the point of fry stocking. All captured fish individuals were identified to a species level, measured and weighed. Fish were identified using the identification key provided by Kottelat and Freyhof [30], and taxonomy followed FishBase [31]. All fish were released back to the rivers as soon as possible after their initial analysis. Sampling was carried out under the permits obtained from the Environment Protection Agency, Lithuania.

Fish abundance (A, ind.) and biomass (B, kg) were calculated and extrapolated for 100 m^2^ (ind./100 m^2^ and kg/100 m^2^): The diversity of fish assemblages was calculated using the Shannon–Weiner diversity index:

H′ = −∑P_i_log_2_P_i_

where H′—index of species diversity; S—number of species in a community; P_i_—number of individuals in each (i) species.

### 2.5. Fish Growth

*Salmo trutta* specimens for growth analyses were annually sampled in both studied streams from May to November in 2019–2022. In total, 1348 specimens of *S. trutta* juveniles were sampled. Fish sampling was performed using battery-powered electric fishing gear. In each stream, fishing was performed from the stream mouth to the point where artificially reared specimens were released (Figure 1). Wherever possible, up to 50 specimens of the age 0^+^ and 1^+^ were collected during each sampling event. Up to five sampling events were performed each year to cover different seasons. The number of collected specimens in the studied streams is provided in Table 3. The overall collected number of individuals for the experiment (1348) accounted for less than 1% of the total number of stocked specimens (15,000) during this study.

All captured *S. trutta* specimens were measured to the nearest 1 mm, weighed to the nearest 0.1 g, and transported to the laboratory for further ARS mark detection. Scales of each captured specimen were collected for determining fish age [32], while a pair of otoliths (saggittae) was removed for determining ARS marks.

**Figure 2:**
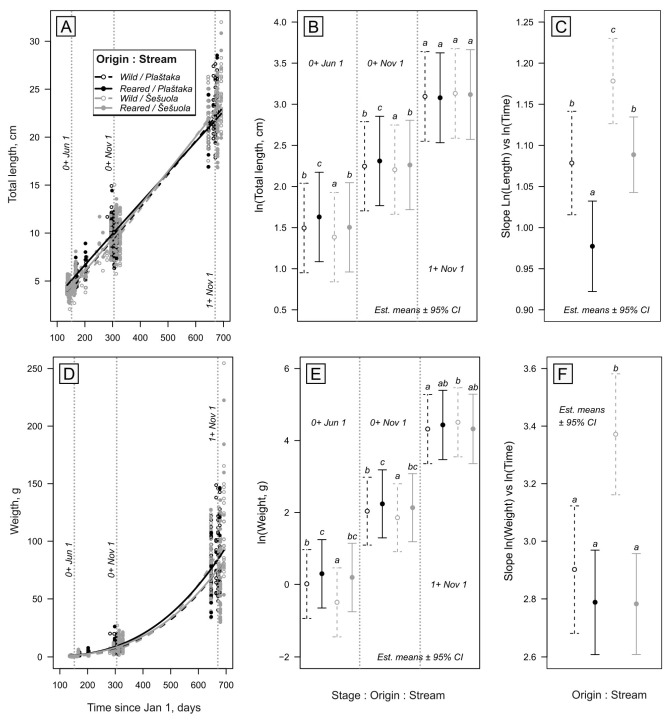
Time-scaled growth (total length and weight) of reared vs. wild *S. trutta* juveniles in studied streams (Šešuola vs. Plaštaka) (see Table 4 for effect tests). (**A**,**D**) depict whole modelled growth curves in the original data scale. Vertical dotted lines in (**A**,**D**) indicate the stages at which the three families of pairwise comparisons were conducted. (**B**,**E**) depict the families of pairwise comparisons within each stage, separated by dotted lines. (**C**,**F**) depict pairwise comparisons of growth slopes. Small letters indicate homogenous groups of estimated means. Note the log-transformed responses in (**B**,**D**) as they were used in statistical analysis.

We modelled the growth of fish as total length and weight functions of time using linear mixed models (LMMs). Both response variables were log-transformed to improve data homoscedasticity, normality, and linearity. The time variable for each cohort (*Days*) was expressed as the number of days since January 1st of the hatching year. This variable was also used log-transformed to improve linearity, and it was then centred around November 1st of the hatching year (day 305, log-transformed) for more meaningful effect testing (Figure 2 depicts original values). The fixed model component included the fully crossed effects of *Time*, *Origin* (reared vs. wild), and *Stream* (Šešuola vs. Plaštaka). The models also included random intercepts for *Year* (2019 to 2022) and *Cohort* (1st to 4th) to account for possible annual climatic and rearing conditions variability. We further proceeded with paired comparisons among each four means estimated (using Satterthwaite degrees of freedom) within three well-covered stages: June 1st and November 1st for age 0^+^ and November 1st for age 1+, as well as for growth slopes for each group. This procedure was conducted with Tukey *p*-value correction for comparing families of four estimates and Šidak correction for confidence levels reflected in visualisation. The analyses were conducted in R v. 4.5.1 environment, employing packages lme4 v.2.0-1 and lmerTest v.3.2-1 (LMM fitting and testing of random effects), performance v.0.16.0 (model assumption inspection), car v.3.1-5 (fixed effect testing), emmeans v.2.0.2 and multcomp v.1.4-30 (pairwise comparisons), and visreg v.2.8.0 (model visualisation).

**Table 4:** Results of LMMs testing the effects of *Time*, *Origin* (reared vs. wild) and *Stream* (Šešuola vs. Plaštaka) on *S. trutta* juveniles’ total length and weight. Significant effects (*p* < 0.05) are in bold.

Term	Total Length		Weight	
	*Χ^2^*	*p*	*Χ^2^*	*p*
Time	**1823.3**	**<0.0001**	**1071.7**	**<0.0001**
Origin	**22.7**	**<0.0001**	**22.9**	**<0.0001**
Stream	**13.9**	**0.0002**	**24.4**	**<0.0001**
Time: Origin	**12.4**	**0.0004**	1.5	0.2243
Time: Stream	**18.8**	**<0.0001**	**37.6**	**<0.0001**
Origin: Stream	0.1	0.7038	1.6	0.2113
Time: Origin: Stream	0.1	0.7351	**17.1**	**<0.0001**

### 2.6. ARS Marks Detection

**Figure 3:**
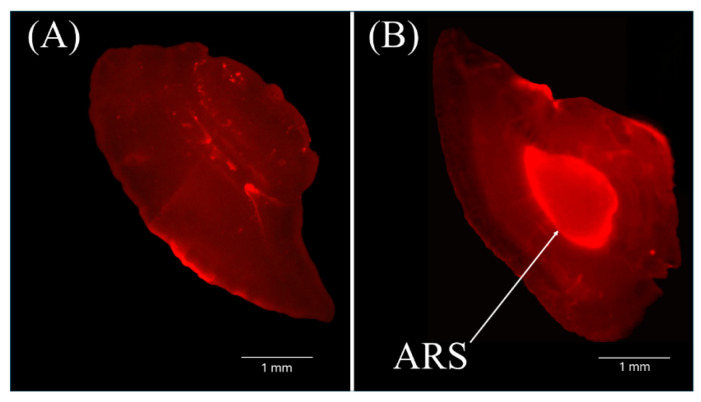
The otoliths (saggittae) of non-marked (**A**) and ARS-marked (**B**) *Salmo trutta* of 1^+^ age in November (2020) under green illumination (4x magnification).

ARS marks detection was performed to separate wild vs. reared *S. trutta* specimens. A pair of otoliths was removed from each of the fish juveniles sampled, then cleaned, desiccated, placed on thin glass slides and kept in the dark until microscopic examination. The presence of ARS marks in fish otoliths was detected under a fluorescence microscope with a magnification power of 4 x. This microscope was equipped with green (λ_ex_ = 560–595 nm and λ_em_ = 645 nm) filters. ARS marks were clearly seen under the fluorescence light (Figure 3).

### 2.7. Fins Damage

**Figure 4:**
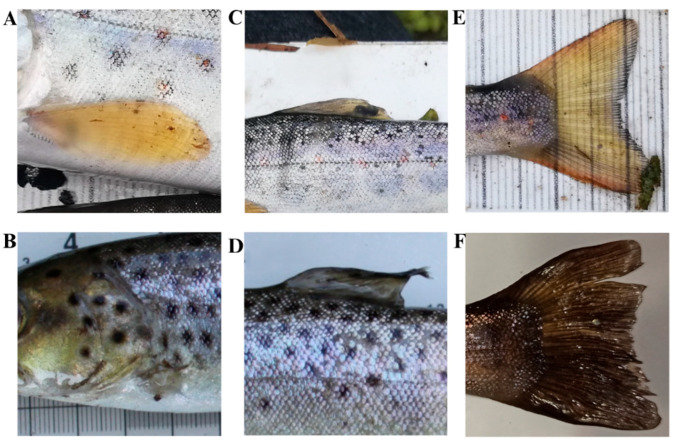
Fins (pectoral, dorsal, caudal) damage of *Salmo trutta* juveniles: intact (**A**,**C**,**E**); reduced or absent (**B**,**D**,**F**).

During the first year of sampling fish, we noticed that fin damage was more frequently expressed within artificially reared *S. trutta* individuals compared to the wild ones. To assess the extent of differences, the fins of the subsample of 699 individuals of *S. trutta* were investigated in 2020–2022. Fin damages were evaluated by visual examination of pectoral, dorsal and caudal fins. Fish were separated into two groups depending on their fin damage: (a) individuals with damaged fins (significantly reduced or absent), and (b) individuals with healthy fins (intact) (Figure 4).

The probability of having a non-intact (reduced or absent) fin was modelled using a general linear mixed model (GLMM) with a binomial (Bernoulli) error distribution, which was built in a manual forward stepwise procedure. The null model included the random intercepts for the specimen ID (1139 levels), River (5 levels), and the Year (2020, 2021, 2022) factors. The scope of fixed terms included a continuous covariate of *Condition* (CF; centred around the mean), and the factors of *Fin location* (caudal, dorsal, pectoral), *Age* (0^+^, 1^+^), *Origin* (reared vs. wild), as well as possible interactions of the four potential predictors. The criteria on subsequent term being included were adherence to the term hierarchy principle, Akaike Information Criterion (AIC) reduction, and a significant model improvement as per the partial *Χ^2^* test. We further proceeded with paired comparisons with Tukey *p*-value correction for comparing families of 12 estimates. The analyses were conducted in R v. 4.5.1 environment, employing packages glmmTMB v.1.1.14 (GLMM fitting), performance v.0.16.0 (model assumption inspection), car v.3.1-5 (fixed effect testing), emmeans v.2.0.2 and multcomp v.1.4-30 (pairwise comparisons).

### 2.8. Haematological Assessment

A significant part of the overall collected fish was used for blood parameters assessment. In total, 456 individuals of artificially reared and wild *S. trutta* juveniles were used. Fish were captured from the natural environment, and blood samples were collected immediately after capture. No additional handling or experimental manipulation was performed prior to blood sampling. These fish were sampled in October 2019, 2020, and 2022 (Table 5). All selected fish were measured and weighed to calculate Fulton’s condition factor (CF). Blood samples were collected to analyse four parameters: number of red blood cells (RBC), the mean cell volume (MCV), the amount of glucose (GLU), and haematocrit value (HCT). The reared fishes were distinguished by the presence of ARS marks in fish otoliths (Figure 3).

A total of 0.2 millilitres of blood was extracted from the caudal vein of the fish using a sterile syringe. The anticoagulant sodium citrate was utilised at a concentration of 3.8%. The blood samples were transferred to blood collection tubes. The determination of glucose concentrations in fish blood was accomplished through the utilisation of the automatic Glucose Analyzer (EKSAN-Gm, Analita, Vilnius, Lithuania). The blood glucose method has a minimum detection limit ranging from 2 to 30 mmol L^−1^, with an error margin for repeated measurements of ≤5%. The measurement of haematocrit was performed using heparinized capillary tubes. The tubes were centrifuged for 5 min at 1500 rpm in a microhematocrit centrifuge (CENTRIFUGE HAEMATOKRIT 210, Hettich, Tuttlingen, Germany). The haematocrit reader was utilised to ascertain the percentage of packed red blood cells. RBC and WBC were counted in a Neubauer chamber using a light microscope, Nikon Eclipse E80i (Nikon Corporation, Tokyo, Japan), in accordance with the method delineated by Ivanova [33]. The neutral red and crystal violet solutions were used as diluting fluids. MCV was calculated according to the method outlined by Haney et al. [34].

**Figure 5:**
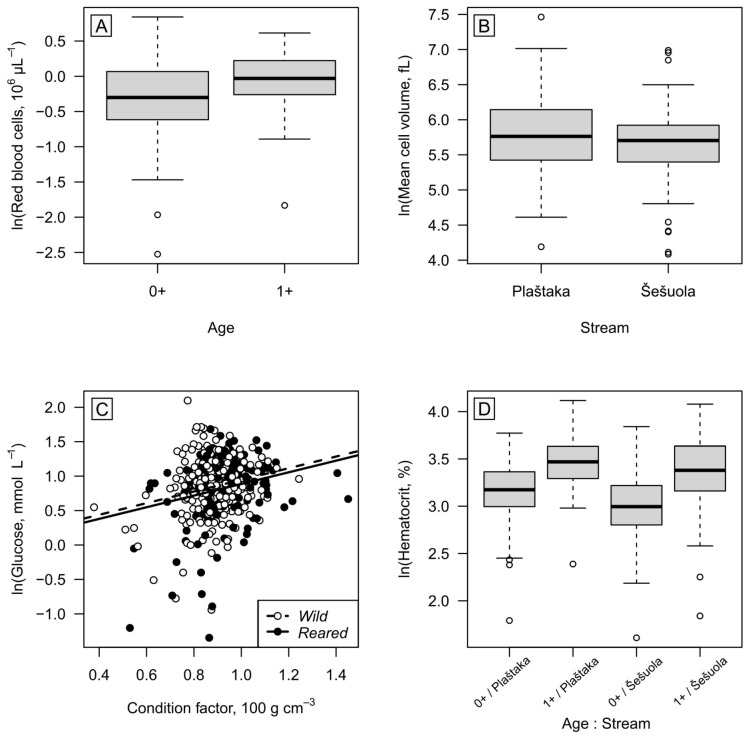
*Condition* (Fulton’s condition factor), *Origin* (reared vs. wild), *Stream* (Šešuola vs. Plaštaka), and *Age* (1^+^ vs. 0^+^) effects on *S. trutta* juveniles’ blood parameters: (**A**): number of red blood cells (RBC), (**B**): the mean cell volume (MCV), (**C**): the amount of glucose (GLU), and (**D**): haematocrit value (HCT). (See Table 6 for effect tests).

We analysed the log-transformed blood parameters (RBC, MCV, GLU, and HCT) as potential functions of *Condition* (CF), *Origin* (reared vs. wild), *Stream* (Šešuola vs. Plaštaka), and *Age* (1^+^ vs. 0^+^), and all possible interactions, using a manual forward stepwise procedure in extending LMMs. The null models only included a random intercept for the *Year* factor (2019, 2020, 2022). The *Condition* covariate was centred around the global mean value (Figure 5 depicts original values). At each step, the most informative candidate term to include would be picked as the one that, when included, would reduce the model AIC value the most. However, the term would only be included in the model if it significantly improved it (as judged by comparing the models with/without the term using the function Anova). During this process, we adhered to the interaction hierarchy principle by not including higher-order interactions without first including all their subcomponents. The analyses were conducted in an R v. 4.5.1 environment, employing packages lme4 v.2.0-1 and lmerTest v.3.2-1 (LMM fitting and testing of random effects), performance v.0.161.0 (model assumption inspection), and car v.3.1-5 (fixed effect testing).

**Table 6:** Results of forward-stepwise LMMs testing the effects of *Condition* (Fulton’s condition factor), *Origin* (reared vs. wild), *Stream* (Šešuola vs. Plaštaka), and *Age* (1^+^ vs. 0^+^) on *S. trutta* juveniles’ blood parameters: number of red blood cells (RBC), the mean cell volume (MCV), the amount of glucose (GLU), and haematocrit value (HCT). Significant effects (*p* < 0.05) are in bold.

Term	RBC		MCV		GLU		HCT	
	*Χ* ^2^	*p*	*Χ* ^2^	*p*	*Χ* ^2^	*p*	*Χ* ^2^	*p*
Condition					**14.8**	**0.0001**		
Origin					**5.8**	**0.0161**		
Stream			**11.2**	**0.0008**			**89.8**	**<0.0001**
Age	**54.5**	**<0.0001**					**21.7**	**<0.0001**

### 2.9. Cultivable Gut Bacteria

A representative part of the overall collected fish was used for a gut bacteria cultivation study. A total of 40 *S. trutta* juveniles of age 0^+^ (20 wild and 20 reared) at the end of October 2019 from the Plaštaka and Šešuola streams were used for analysis of cultivable gut bacteria. Selected specimens were placed in sterile plastic bags and transported on ice to the laboratory. Fish were aseptically dissected, and the contents of the midgut and distal intestine were collected. The gut contents of 3–4 fish from one tested group were pooled to minimise individual variation in gut bacteria, and then serially diluted (1:10) in Phosphate Buffered Saline (PBS, pH 7.3, Oxoid, Hampshire, UK). 100 µL of each dilution (10^−4^ to 10^−8^) was spread onto Tryptone Soya Agar (TSA, Oxoid, Hampshire, UK) plates in triplicate. The plates were incubated at 15 °C for 96 h, after which the colony-forming units (CFU/g) were calculated and expressed as Log_10_ CFU/g (mean ± SD).

The assessment of cultivable gut bacteria data was conducted using a one-way ANOVA, followed by a Bonferroni post hoc test using Statistica 7.0 software (USA). Differences were accepted as significant at the 95% level of confidence (*p* < 0.05).

## 3. Results

### 3.1. Streams Water Temperature

The measured annual water temperature averages showed a clear annual pattern in the differences in water temperatures of the studied streams. Results revealed a notably higher (from 1.46 to 1.84 °C) measured average of the annual temperature values in Šešuola Stream compared to the Plaštaka (Table 7). The difference can be explained by the artificial dam in the upper part of the Šešuola stream, which gives a flow of slightly warmed surface water from the dam pond.

According to Elliott [35], two distinct thermal ranges are critical for the growth dynamics of *S. trutta*. The optimal physiological range, within which the species actively feeds and experiences minimal thermal stress, spans from 4 to 19 °C. However, the range at which energy conversion efficiency is maximised, resulting in peak growth rates, is narrower, extending from 8 to 17 °C. Our thermal data revealed a considerably higher number of days within both intervals in the Plaštaka Stream compared to the Šešuola Stream. Throughout the monitoring period, 440 days in the Plaštaka Stream fell within the optimal physiological range (4–19 °C), whereas only 328 days were recorded in the Šešuola Stream. More strikingly, the Plaštaka Stream provided 172 days within the range required for maximum growth (8–17 °C), compared to only 60 days in the Šešuola Stream.

### 3.2. Fish Assemblages

Fish abundance and biomass in Šešuola Stream (183.1 ind./100 m^2^ and 3.10 kg/100 m^2^) were 3 times higher than in Plaštaka Stream (62.2 ind./100 m^2^ and 1.3 kg/100 m^2^), respectively. In the Šešuola stream, 16 different fish species were caught during the fish assemblage’s assessment, compared to 20 species in the Plaštaka Stream (Table 8). In the Šešuola Stream, fish from the family Leuciscidae dominated the fish assemblage, constituting 39.4% of it. Conversely, in the Plaštaka stream salmonids (Salmonidae) were the dominant group among the whole fish assemblage, reaching 28.0% and the subdominant group Leuciscidae (23.0%). Analysis of biomass distribution revealed that *Perca fluviatilis* was dominant (51.2%) in the Šešuola Stream, followed by the subdominant species *S. trutta* (22.6%). In contrast, the majority of biomass in the Plaštaka stream comprised *S. trutta* (53.8%). Further analysis using the Shannon–Weiner diversity index, despite the smaller number of detected species (16 in Šešuola, 20 in Plaštaka), showed that the Šešuola stream (H′ = 2.09) had a higher index value compared with the Plaštaka stream (H′ = 1.74).

### 3.3. S. trutta Growth

Initially, the reared *S. trutta* fry were significantly larger than the naturally hatched fry present in the studied streams at that time. The artificially reared and stocked fry were up to 36% longer and up to 75% heavier (Table 9).

Further *S. trutta* growth analysis also revealed that *Origin* (reared vs. wild) remains a significant factor when considering fish length and weight (Table 4). This trend is clearly seen, especially during their first year. Artificially reared individuals were significantly bigger after their first year (Figure 2). However, differences vanished while reaching their second year, just before smoltification (Figure 5). This indicates a slower growth of reared fry compared to the natural ones. Results also revealed that the *Stream* factor was significant when considering fish weight and length (Table 4). Fishes were slightly bigger in the Plaštaka Stream compared to the Šešuola Stream, especially during their first year (Figure 4).

### 3.4. Fins Damages

An assessment of fin damage revealed significantly higher prevalence among artificially reared *S. trutta* juveniles (Table 10). Overall, fin damage was observed in 75% of artificially reared 0^+^ *S. trutta* juveniles, while only 24% of wild specimens had damaged fins. The same trend was observed in older (1^+^) individuals (Table 10).

**Figure 6:**
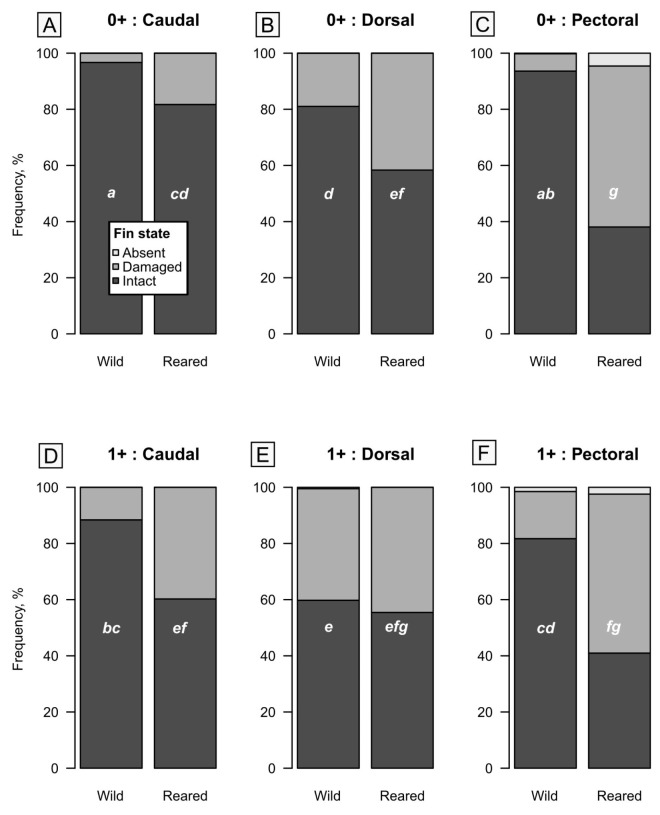
Raw total frequencies of fin state by fin location (left column: caudal (**A**,**D**), middle column: dorsal (**B**,**E**), right column: pectoral (**C**,**F**), age (top row: 0^+^ (**A**–**C**), bottom row: 1^+^ (**D**–**F**) and origin (wild, reared) of *S. trutta* juveniles. The small white letters indicate homogenous groups of estimated means in pairwise comparisons (see Table 11 for effect tests; damaged and absent fins were analysed jointly).

Statistical analyses showed that fish *Origin* (reared vs. wild) was significant for the observed frequency of fin damage (Table 11). Interestingly, the fins’ position (caudal, dorsal or pectoral) was also crucial for the frequency of damage (Table 11). Among reared juveniles, the most often damaged were observed within pectoral fins, while dorsal fin damage was the most frequent among wild individuals. Caudal fin damage was the rarest among both reared and wild juveniles (Table 11, Figure 6). Surprisingly, the age factor was also significant for the fin’s damage (Table 11). First-year wild juveniles had fewer damage counts compared to the second-year juveniles (Table 10, Figure 6). However, such a trend was not observed for the reared juveniles. More than 70% of reared individuals had fin damage among both 0^+^ and 1^+^ juveniles (Table 10). The forward-stepwise GLMM indicated that up to 2nd order interaction between tested factors had a significant effect on the probability of fin damage, while the Condition factor was not selected for the model.

### 3.5. Haematological Analyses

Haematological analyses revealed that *Origin* (reared vs. wild) was an important factor only for the glucose concentration in fish blood. Results also showed that fish age significantly increased the number of RBC and the value of HCT in the studied fish blood. At the same time, the *Stream* effect was significant for the HCT values and the MCV (Table 6). These parameters were higher in Plaštaka Stream (Figure 6). Blood analyses also showed that higher values of fish overall fitness (*Condition* factor) are related to higher glucose concentrations in fish blood (Table 6, Figure 5).

### 3.6. Cultivable Gut Bacteria

Analysis of *S. trutta* juvenile gut bacteria showed that the number of cultivable bacteria in reared fish from the Šešuola and Plaštaka streams five months after stocking was significantly higher than in wild fish (one-way ANOVA: Šešuola F_2.10_ = 49.00; *p* < 0.005 and Plaštaka F_2.13_ = 86.018; *p* < 0.005, respectively) (Table 12).

## 4. Discussion

### 4.1. Basic Environment Conditions

Generally, the growth of *S. trutta* is profoundly influenced by a complex interplay of abiotic and biotic factors, including water temperature [36,37], dissolved oxygen levels [38,39], prey availability [40,41], competition for food and shelter [42,43,44,45], and genetic lineage [46,47,48]. The two streams investigated in this study exhibited similarities in catchment area, mean annual flow, and hydrological regime. Furthermore, the genetic background of the *S. trutta* juveniles was likely comparable, as the streams are geographically proximate and belong to the same population within the Siesartis River basin; notably, the parental stock for the artificially reared fry was also sourced from the Siesartis River. Despite these commonalities, the streams differed significantly in their thermal profiles and biological productivity. The Šešuola Stream was characterised by higher mean temperatures and a three-fold increase in overall fish abundance and biomass compared to the Plaštaka Stream, suggesting a higher trophic status. Conversely, the Plaštaka Stream maintained a significantly higher number of days within the optimal temperature range for *S. trutta* growth throughout the study period. Additionally, interspecific competition was likely less intense in the Plaštaka Stream. These environmental and community disparities may account for the observed differences in S. trutta juvenile growth between the two sites.

### 4.2. Fins Damage

A significant increase in fin damage was observed among hatchery-reared *S. trutta* specimens compared to their wild counterparts. Such a high prevalence of fin erosion is a well-documented phenomenon in artificially reared salmonids [49,50,51,52,53], with some researchers even suggesting that fin degradation may serve as a reliable indicator of hatchery origin [54]. However, our findings demonstrate that fin damage also occurs in wild populations, albeit to a lesser extent. Generally, fin injuries arise from a variety of stressors, including abrasive surfaces, agonistic interactions, nutritional imbalances, high stocking densities, poor water quality, and bacterial infections [55,56,57]. Specifically, dorsal fins are most frequently injured during aggressive encounters, whereas pectoral fin damage predominantly results from contact with tank walls. In contrast, anal and pelvic fins are less prone to agonistic damage but remain susceptible to abrasion from benthic substrates [56,57]. The primary concerns regarding fin degradation involve the subsequent survival and performance of fish in natural environments [51]. Severe fin damage impairs swimming ability, potentially reducing a fish’s capacity to adapt and survive in the wild, and is often correlated with diminished growth and higher mortality rates [58,59].

Our findings indicate that pectoral fin damage was the most prevalent injury among hatchery-reared juveniles, whereas dorsal fin damage was most frequent in wild individuals, albeit with a significantly lower overall prevalence compared to the hatchery group. Caudal fin injuries were the least common in both cohorts. This pattern of fin degradation aligns with the established understanding that pectoral fin trauma primarily results from abrasion against tank walls in artificial environments, while dorsal fin damage typically occurs during agonistic interactions in the wild [56,57].

In summary, fin damage in *S. trutta* juveniles likely impairs foraging efficiency, increases metabolic costs associated with tissue repair, and facilitates secondary infections, all of which contribute to reduced growth rates [60,61,62]. Consequently, substantial fin erosion or loss may be a critical factor limiting the migratory capacity and, ultimately, the long-term reproductive success of hatchery-reared *S. trutta* juveniles.

### 4.3. Fish Growth

It is well-established that hatchery-reared fish may initially retain a size advantage upon introduction into natural environments, despite potential physiological maladaptation, impaired swimming performance, or behavioural deficits often associated with a high prevalence of fin damage. Indeed, some studies suggest that the combination of greater body length and mass, alongside characteristic fin erosion, serves as a reliable metric for distinguishing aquaculture-reared salmonids from their wild counterparts [54,63]. However, this initial advantage in size and weight is frequently transient; evidence suggests that reared individuals often lose their competitive edge over time when subjected to the rigours of natural environments [64].

Our results demonstrate that hatchery-reared *S. trutta* juveniles were considerably larger than naturally hatched individuals during their first year. This initial disparity stems from the fact that the reared fry was significantly larger (up to 36% longer) than their wild counterparts at the time of stocking, a result of the stable, optimal thermal conditions and consistent food availability provided during artificial rearing. This pattern aligns with documented trends in aquaculture, where reared fish typically exhibit accelerated growth and reach greater sizes than wild fish of the same age [63]. However, our findings indicate that these individuals lost this initial size and weight advantage over time following their release. By the end of the second year, immediately prior to smoltification and seaward migration, the previously observed size differences had dissipated in both streams (Figure 4), regardless of variations in thermal regimes or productivity. This suggests that hatchery-reared *S. trutta* juveniles exhibit slower growth rates than naturally hatched individuals under wild conditions. Several factors may contribute to this growth retardation. Post-stocking, artificially reared fry may face significant challenges in adapting to natural environments [8,9,10,11,12,13], potentially losing the competition for critical resources, such as food and habitat, to naturally spawned residents [14,15]. Furthermore, the high prevalence of fin damage observed in this study likely exerted a negative influence on the growth performance of the reared fish.

Overall, within a two-year period, the wild *S. trutta* juveniles—despite their significantly smaller initial size—attained dimensions comparable to those of their hatchery-reared counterparts. By the onset of their primary seaward migration to the Baltic Sea, no significant disparities in size or physiological fitness were observed between the two groups. These findings indicate that the post-release growth rate of hatchery-reared juveniles is markedly lower than that of wild individuals under natural conditions. Should this suboptimal growth trajectory persist into later life stages, it may suggest that artificially reared specimens possess a reduced probability of reaching maturity and achieving successful recruitment.

When interpreting these results, it is crucial to consider the genetic architecture of the stocked cohorts. Notably, within each study year, the artificially reared individuals originated from a single breeding pair, a factor that may have constrained genetic variability and, consequently, adaptive potential. However, as the study spanned four consecutive years and utilised different parental pairs for each annual release, the observed phenotypic patterns remained consistent across multiple independent cohorts. This longitudinal consistency suggests that the disparities between reared and wild juveniles likely reflect the synergistic effects of artificial rearing environments and cohort-specific genetic constraints, rather than being an artefact of a single parental effect.

These findings are consistent with recent literature indicating that hatchery-reared salmonids frequently exhibit altered growth trajectories and diminished ecological performance upon introduction to natural environments. Previous syntheses have demonstrated that such disparities typically arise from a synergy of genetic and environmental factors inherent to hatchery rearing [16]. Furthermore, the efficacy of stocking programmes has proven to be highly variable, contingent upon both the specific ecological context and the overarching management strategies employed [18]. Experimental evidence further suggests that environmental conditions during early life stages can exert long-lasting effects on the growth, behaviour, and adaptive capacity of salmonids [65]. This supports the interpretation that the differences observed in the present study reflect complex developmental and ecological processes, rather than being attributable to a single causal factor.

### 4.4. Physiological Condition

Generally, haematological parameters—including red blood cell count (RBC), mean corpuscular volume (MCV), glucose (GLU) concentration, and haematocrit (HCT) levels—serve as robust indicators of overall fish health and physiological fitness [66,67]. Even subtle haematological fluctuations can function as early warning signals of subclinical stress, particularly in environments characterised by complex stressors [68]. The results of this study demonstrate that the selected blood parameters were primarily influenced by the age of the *S. trutta* juveniles and the specific characteristics of their stream habitat. Notably, fish origin (wild versus hatchery-reared) had a significant impact only on GLU concentration, suggesting that while growth and morphology differ between the cohorts, their broader physiological profiles are largely shaped by shared environmental conditions.

Glucose is one of the most rapidly responding and frequently measured indicators of physiological stress in fish. However, there is a lack of data comparing GLU levels in reared and wild fish. Typically, glucose levels are investigated in farmed fish. Han et al. [69] demonstrated that *O. niloticus* fed high-starch diets exhibited concurrent increases in fitness and GLU. Similarly, Callet et al. [70] showed that rainbow trout fed low-protein, high-carbohydrate diets developed altered GLU metabolism, including increased glycogen storage and hyperglycaemia. Furthermore, GLU levels vary by species, environment, toxicant type, and exposure duration, complicating interpretation [71]. Acute stressors such as handling, hypoxia, or pollutants have been shown to rapidly induce hyperglycaemia by activating secondary stress pathways [72,73]. Ma et al. [74] and Biswal et al. [72] demonstrated that the presence of stress-induced increases in GLU activates glycolytic and other stress-related metabolic pathways. Glucose is widely used as an indicator of physiological stress and energy status; however, Witeska et al. [75] noted that it should be interpreted within a broader haematological context to avoid false positives. Similarly, Krishnan and Rohner [76] emphasise that teleost models of hyperglycaemia demonstrate high plasticity in GLU regulation, particularly under nutritional or environmental stress. In our study, elevated GLU levels were observed in reared juveniles compared to wild individuals, without corresponding changes in RBC or haematocrit. This pattern suggests differences in metabolic status between reared and wild juveniles, potentially related to rearing history. However, given the non-specific nature of glucose as a biomarker, these differences cannot be attributed to a single underlying cause.

It is well-established that fish erythrocyte indices serve as sensitive indicators of changes in water quality and environmental factors. Studies have shown that MCV, RBC parameters, and HCT vary under different environmental conditions [77,78]. MCV reflects erythrocyte size and is commonly associated with oxygen transport capacity and metabolic activity in fish [75,78]. Lower MCV values were observed in fish from the Plaštaka Stream than in fish from the Šešuola Stream. This inter-river difference may be related to environmental variability, including differences in temperature regimes, oxygen availability, and metabolic demands. Variations in MCV have been linked to environmental conditions and physiological adjustments in fish, although the underlying mechanisms are often complex and species-specific [66,76,78,79]. Therefore, while the observed differences in MCV are plausibly linked to environmental variation between streams, their physiological significance remains complex and likely reflects multiple interacting environmental and biological factors.

The HCT values observed in this study are consistent with those previously reported in the literature for salmonid species. For example, Nabi et al. [80] reported HCT values ranging from 29% to 40% in *Oncorhynchus mykiss* reared under standardised Himalayan aquaculture conditions. Sheikha et al. [81] found average HCT levels of 35% in *S. trutta*, with no significant difference between the sexes. The age-related increase in HCT observed in our study aligns with well-documented physiological changes that occur during salmonid growth [78]. As fish grow, HCT increases due to rising metabolic demands and increased erythrocyte production [82,83]. This trend is reflected in haematological reference values for *O. mykiss*; juvenile fish typically have a lower HCT (31%) than adult fish (37%) [80]. Additionally, differences in HCT values may be determined by habitat-specific metabolic demands [83,84]. The significant stream effect observed in our study may be explained by differences in environmental oxygen availability, water temperature, or cumulative exposure to stressors. These factors influence erythropoietic output and blood viscosity regulation.

Our haematological analysis provides novel insights by comparing wild and hatchery-origin juveniles under natural conditions, demonstrating that physiological indicators, such as glucose, may not directly correspond to growth trajectories or overall fitness outcomes. Consistent with this, our findings indicate that both environmental variables and rearing origin contribute to observed physiological disparities, with their respective influences being modulated by stream-specific conditions. Collectively, these results underscore the necessity of interpreting haematological responses within a broader ecological context, as physiological markers are shaped by a complex interplay between an individual’s ontogeny and its immediate environment.

### 4.5. Cultivable Gut Bacteria

The fish gut microbiota is fundamental to host health, facilitating nutrient degradation and absorption, modulating immune system development, and synthesising essential vitamins and bioactive molecules. Furthermore, it serves as a critical line of defence against pathogens by maintaining intestinal barrier integrity [85,86,87]. Previous research has identified statistically significant disparities in microbiota composition between wild and hatchery-reared fish [88,89,90,91]. Interestingly, species richness and diversity in the gut microbiota of wild *Salmo salar* juveniles have been found to be significantly lower than in hatchery-reared individuals [88,92]. Given that hatchery-origin fish often exhibit lower post-stocking survival rates [93,94,95], the transition to natural environments likely imposes significant physiological stress. Such environmental stressors can detrimentally impact the health and fitness of salmonid fry by inducing dysbiosis in the gut microbiome [96,97]. Longitudinal studies (e.g., two months post-release) comparing *S. salar* parr from the same genetic population have shown that gut microbiota remains significantly different and is strongly influenced by the early rearing environment [92]. Our own preliminary findings align with this, revealing significant differences in the gut microbiota of *S. trutta* juveniles between wild and hatchery-reared groups. Five months post-release, the abundance of culturable (viable) bacteria in the gut of hatchery-reared fish remained significantly higher than in their wild counterparts across both studied streams. These microbial disparities could potentially confer phenotypic disadvantages, such as reduced disease resistance and impaired metabolic efficiency [98,99,100]. However, further research is required to characterise the predominant bacterial taxa in hatchery-reared *S. trutta* over longer post-stocking periods and to evaluate their specific impact on juvenile growth and overall fitness.

## 5. Conclusions

This study demonstrates that although hatchery-reared *S. trutta* juveniles do not significantly differ from wild individuals in terms of size or haematological parameters after two years in a natural environment, they exhibit slower growth rates and a higher prevalence of fin damage, both of which may adversely impact their subsequent life cycle. These findings suggest that fin erosion, coupled with a potentially protracted or less effective adaptation to wild conditions, constitutes a primary driver of the observed growth retardation. Furthermore, the fish gut microbiome appears to be significantly associated with the growth and physiological condition of reared individuals, as marked disparities in microbial composition were observed between wild and artificially reared juveniles. Longitudinal studies encompassing later life stages are essential to identify the decisive factors influencing long-term performance and to determine the extent to which these early-life physiological and ecological constraints manifest in adult populations.

## Figures and Tables

**Table 1:** Hydrological characteristics of the streams studied [23].

Stream	Length (km)	Catchment Size (km^2^)	Mean Annual Flow (m^3^s^−1^)	Average Slope in the Lower Reaches (3.0–3.5 km to the Mouth), (km^−1^)	Mean Width, (m)	Mean Depth, (m)
Plaštaka	16.1	89.3	0.67	6.0	3.6	0.3
Šešuola	13.6	91.2	0.64	6.1	4.3	0.4

**Table 2:** Stocked *S. trutta* specimens in different streams and years: number of stocked specimens (N); fish total length (TL, cm); fish total weight (Q, g).

Year	N (Šešuola)	N (Plaštaka)	TL (Mean ± SD)	Q (Mean ± SD)	Release Date
2019	1500	1500	3.63 ± 0.22	0.40 ± 0.07	20 May 2019
2020	2000	2000	5.38 ± 0.57	1.40 ± 0.41	21 May 2020
2021	2000	2000	4.11 ± 0.28	0.67 ± 0.19	20 May 2021
2022	2000	2000	4.97 ± 0.35	1.10 ± 0.23	20 May 2022

**Table 3:** Numbers of sampled *S. trutta* in studied streams 2019–2022 for growth assessment: sampling year; number of sampled wild vs. reared specimens of age 0^+^ and 1^+^.

River	Year	0^+^ (Wild/Reared)	1^+^ (Wild/Reared)
Šešuola	2019	275 (90/185)	-
	2020	157 (86/71)	56 (47/9)
	2021	98 (68/30)	16 (16/0)
	2022	107 (56/51)	61 (25/36)
Plaštaka	2019	52 (38/14)	-
	2020	116 (51/65)	49 (42/7)
	2021	116 (64/52)	14 (13/1)
	2022	184 (94/90)	47 (37/10)

**Table 5:** Numbers of sampled *S. trutta* in studied streams 2019–2022 for blood parameters assessment: sampling year; number of sampled wild vs. reared specimens of age 0^+^ and 1^+^.

Stream	Year	0^+^ (Wild/Reared)	1^+^ (Wild/Reared)
Šešuola	2019	31/17	-
	2020	24/20	47/9
	2022	9/26	3/32
Plaštaka	2019	36/12	-
	2020	22/28	43/7
	2022	40/25	19/6

**Table 7:** The annual averages of measured water temperatures (T, °C) in the studied Šešuola and Plaštaka streams.

Year	Šešuola, (Mean ± SD)	Plaštaka (Mean ± SD)
2019	14.71 ± 3.71	12.87 ± 3.19
2020	14.18 ± 5.18	12.72 ± 4.65
2022	15.27 ± 5.45	13.64 ± 5.32

**Table 8:** Fish species abundance (A, ind./100 m^2^), and percentage biomass (B, kg/100 m^2^) in studied streams.

No	Fish Species	Šešuola		Plaštaka	
		A	B	A	B
1	*Abramis brama*	0.7	0.1	0.0	0.0
2	*Alburnoides bipunctatu*	0.0	0.0	2.5	< 0.1
3	*Alburnus alburnus*	0.3	<0.1	2.5	<0.1
4	*Anguilla Anguilla*	0.0	0.0	0.2	0.2
5	*Barbatula barbatula*	15.7	0.1	1.6	<0.1
6	*Cobitis taenia*	0.3	<0.1	0.2	<0.1
7	*Cottus gobio*	6.7	0.1	3.6	<0.1
8	*Esox lucius*	0.3	0.1	0.2	0.1
9	*Gasterosteus aculeatus*	0.3	<0.1	0.2	<0.1
10	*Gobio gobio*	22.7	0.2	0.4	<0.1
11	*Leuciscus leuciscus*	0.3	<0.1	0.2	<0.1
12	*Misgurnus fossilis*	0.0	0.0	0.2	<0.1
13	*Perca fluviatilis*	25.3	1.3	2.0	<0.1
14	*Phoxinus phoxinus*	33.3	0.3	12.2	0.1
15	*Rhodeus sericeus*	16.0	<0.1	2.4	<0.1
16	*Rutilus rutilus*	36.3	0.2	4.7	0.1
17	*Salmo salar*	0.3	<0.1	0.2	<0.1
18	*Salmo trutta*	23.3	0.7	27.6	0.7
19	*Squalius cephalus*	1.3	<0.1	0.9	0.1
20	*Thymallus thymallus*	0.0	0.0	0.2	<0.1
21	*Tinca tinca*	0.0	0.0	0.2	<0.1
	Total	183.1	3.1	62.2	1.3

**Table 9:** Mean values of total length and weight of reared vs. wild *Salmo trutta* fry during the stocking period in 2019–2022. Statistical differences (*p*-value, Man-Whitney U-test) between reared and wild fry.

Stream	Year	Reared	Wild	*p* Value
Total length (cm)				
Plaštaka	2019	3.63 ± 0.22	3.10 ± 0.25	<0.001
Plaštaka	2020	5.38 ± 0.57	4.79 ± 0.67	0.032
Šešuola	2019	3.63 ± 0.22	3.48 ± 0.26	0.030
Šešuola	2020	5.38 ± 0.57	3.44 ± 0.56	<0.001
Šešuola	2022	4.97 ± 0.35	3.96 ± 0.54	<0.001
Weight (g)				
Plaštaka	2019	0.40 ± 0.07	0.21 ± 0.27	<0.001
Plaštaka	2020	1.40 ± 0.41	1.08 ± 0.44	0.025
Šešuola	2019	0.40 ± 0.07	0.32 ± 0.09	0.006
Šešuola	2020	1.40 ± 0.41	0.33 ± 0.27	<0.001
Šešuola	2022	1.10 ± 0.23	0.54 ± 0.23	<0.001

**Table 10:** Frequency of fin damage among reared and wild *S. trutta* juveniles. Statistical differences (Chi square, *p*-value) between reared and wild juveniles.

Fin	Reared	Wild	*p* Value
0^+^	N = 142	N = 314	
Pectoral	85 (59.9%)	14 (4.5%)	<0.001
Dorsal	49 (34.5%)	62 (19.7%)	<0.001
Caudal	34 (23.9%)	7 (2.2%)	<0.001
Total	107 (75.4%)	74 (23.6%)	<0.001
1^+^	N = 63	N = 180	
Pectoral	33 (52.4%)	27 (15.0%)	<0.001
Dorsal	30 (47.6%)	57 (31.7%)	0.023
Caudal	22 (34.9%)	8 (4.4%)	<0.001
Total	45 (71.4%)	70 (38.9%)	<0.001

**Table 11:** Results of forward-stepwise GLMM testing the effects of *fin location* (pectoral, dorsal, caudal), *Age* (0^+^ vs. 1^+^), and *Origin* (reared vs. wild) effects on the frequency of fin damage in *S. trutta* juveniles. Significant effects (*p* < 0.05) are in bold.

Term	*Χ^2^*	*Df*	*p*
Fin location	**68.5**	**2**	**<0.0001**
Age	**15.6**	**1**	**0.0001**
Origin	**34.4**	**1**	**<0.0001**
Fin location: Origin	**54.5**	**2**	**<0.0001**
Fin location: Age	0.0	2	0.9783
Age: Origin	0.0	1	0.9344
Fin location: Age: Origin	**6.5**	**2**	**0.0380**

**Table 12:** Number (Log_10_ CFU/g) of cultivable gut bacteria in *Salmo trutta* juveniles from the Šešuola and Plaštaka streams in 2019, five months after stocking.

Stream	Reared	Wild
Šešuola	7.3 ± 0.1	6.9 ± 0.1
Plaštaka	7.7 ± 0.1	7.1 ± 0.1

## Data Availability

The raw data supporting the conclusions of this article will be made available by the authors on request.

## Abbreviations

The following abbreviations are used in this manuscript:

ARS	Alizarin Red S
CF	Fulton’s condition factor
RBC	Number of red blood cells
MCV	The mean cell volume
GLU	The amount of glucose
HCT	Haematocrit value
